# Revised recommendations of the Italian Society of Pediatrics about the general management of Kawasaki disease

**DOI:** 10.1186/s13052-021-00962-4

**Published:** 2021-01-25

**Authors:** Alessandra Marchesi, Donato Rigante, Rolando Cimaz, Angelo Ravelli, Isabella Tarissi de Jacobis, Alessandro Rimini, Fabio Cardinale, Marco Cattalini, Andrea De Zorzi, Rosa Maria Dellepiane, Patrizia Salice, Aurelio Secinaro, Andrea Taddio, Paolo Palma, Maya El Hachem, Elisabetta Cortis, Maria Cristina Maggio, Giovanni Corsello, Alberto Villani

**Affiliations:** 1 Bambino Gesù Children’s Hospital, Rome, Italy; 2 Department of Life Sciences and Public Health, Fondazione Policlinico A. Gemelli IRCCS, Rome, Italy; 3 Università Cattolica Sacro Cuore, Rome, Italy; 4 Pediatric Rheumatology, ASST Gaetano Pini-CTO, Milan, Italy; 5 University of Milan, Milan, Italy; 6 Pediatrics and Rheumatology Unit, IRCCS Istituto Giannina Gaslini, University of Genoa, Genoa, Italy; 7 IRCCS Istituto Giannina Gaslini, Genoa, Italy; 8 Department of Pediatrics, AOU Policlinico Giovanni XXIII, Bari, Italy; 9 Pediatrics Clinic, University of Brescia, ASST Spedali Civili di Brescia, Brescia, Italy; 10 UOC of Pediatrics, UOS of Pediatric Immunology, Fondazione IRCCS Ca’ Granda Ospedale Maggiore Policlinico, Milan, Italy; 11 Section of Pediatric Cardiovascular Diseases, Fondazione IRCCS Ca’ Granda Ospedale Maggiore Policlinico, Milan, Italy; 12 University of Trieste, Trieste, Italy; 13 Institute of Child and Maternal Health, IRCCS Burlo Garofolo, Trieste, Italy; 14 UOC of Pediatrics, S. Eugenio Hospital, Rome, Italy; 15 Università degli Studi di Palermo, Palermo, Italy

**Keywords:** Kawasaki disease, Coronary artery abnormalities, Intravenous immunoglobulin, Aspirin, Children

## Abstract

Aim of these revised recommendations for the general management of Kawasaki disease is to encourage its prompter recognition and warrant the most appropriate therapy, based on ascertained scientific data, raising awareness of the complications related to misdiagnosis or delayed treatment. A set of 20 synthetic operative statements is herein provided, including the definition of Kawasaki disease, its protean presentations, clinical course and seminal treatment modalities of all disease phases. The application of these recommendations should improve prognosis of Kawasaki disease and prevent the progression to permanent vascular abnormalities, thereby diminishing morbidity and mortality.

## Background

Two years after the diffusion of the Italian Guidelines for diagnosis and treatment of Kawasaki disease (KD), we have updated 20 operative recommendations focusing on the general management of this widespread illness of unknown etiology, which mostly occurs in infants and children under 5 years of age with risk of heart implications, especially if the diagnosis is missed or if timely appropriate treatment is overlooked [1, 2]. First described in Japan in l967 by Tomisaku Kawasaki, the disease occurs in children of all ethnicities and is one of the leading cause of acquired cardiovascular disease in the pediatric age [3]. The basic symptom of KD is persistently high and nonresponding fever, despite antipyretic and antibiotic treatments [4]. Japan, Korea and Taiwan are the countries with the highest frequency of KD, reflecting a potential genetic susceptibility among the Asian populations. In addition, the age distribution of KD, higher in children less than 2 years and lower in those less than 6 months, is consistent with a potentially infectious disease, though KD has been even reported in newborns [5, 6].

The identification of the causing mechanisms behind KD would be essential to develop preventive strategies or focused therapies, but the definite etiology remains elusive. A number of theories have linked KD to bacteria, viruses and different environmental factors, but none has been proved. It is discussed if the trigger of KD might be an RNA virus that normally causes an asymptomatic infection in most children and KD in a subset of genetically predisposed children [7]. The most significant complications in KD are cardiovascular and prominently involve coronary arteries, though the overall incidence of coronary artery abnormalities (CAA) has been reduced by treatment with intravenous immunoglobulin (IVIG) given within 7–10 days after disease onset [8]. Diagnosis of KD is *clinical,* supported by the results of labworks and imaging, but no findings or test can be considered highly specific. Diagnostic difficulties depend also on the different presenting times of signs and symptoms, the gruelling identification of other infectious and non-infectious illnesses mimicking KD, the occurrence of non-typical manifestations and incomplete patterns of the disease [9, 10].

Aim of these synthetically revised recommendations is suggesting the best practice for the management of children with KD, based on the most recent scientific evidence.

### Definitions of Kawasaki disease

#### Typical KD

Typical KD is characterized by fever lasting at least 5 days combined with at least 4 among 5 main clinical features (*clinical criteria*):

[a] bilateral non-exudative conjunctivitis,

[b] anomalies of the lips and oral mucosa (redding and cracking of lips, strawberry tongue, pharyngeal hyperemia),

[c] anomalies of the extremities (redding of palms and soles, indurative edema of hands and feet) and perineal region,

[d] polymorphous exanthema,

[e] acute nonpurulent cervical lymphadenopathy (usually unilateral and larger than 1,5 cm).

The suspicion of KD may be initially cumbersome, as clinical features do not appear at the same time [11]. The presence of fever is universal and fever onset is considered the first day of the illness, though the diagnosis of KD should be considered also in children in whom fever has subsided before the 5th day [12].

The 2017 American Heart Association guidelines suggest that in the presence of more than 4 main clinical features, particularly when the child shows redness and swelling of hands and feet, the diagnosis can be established with only 4 days of fever. They also claim that experienced clinicians who have treated many KD patients could establish a diagnosis even with only 3 days of fever in the presence of classic clinical features [13].

The decision to hospitalize or discharge children with fever lasting around 5 days might depend on the educational and socioeconomic status of their families or the availability of community medical services [14]. Several children may have incomplete or atypical forms of KD and discrimination of such forms from infections and other febrile conditions of childhood is an arduous challenge even for an experienced pediatrician: scholastically, incomplete KD should be considered in case of children with unexplained fever for more than 5 days associated with 2 or 3 classic features, while atypical KD in case of children with unexplained fever for more than 5 days associated with non-classic clinical signs which do not respond to a proper therapy. The most fearful complications of KD are CAA, which develop in 15-to-25% of non-treated patients, but only in 5% of those properly treated, and are normally detected by echocardiography [13].

### Recommendation 1

Typical KD is diagnosed when fever lasting more than 5 days is associated with ≥ 4 *clinical criteria* (bilateral non-exudative conjunctivitis, changes of lips and oral mucosa, changes of the extremities and perineal region, polymorphous exanthema and cervical lymphadenopathy); in the case of CAA early demonstrated on echocardiography diagnosis of KD is possible also when a 4-day-lasting fever is associated with the known criteria.

#### Incomplete KD

The medical literature has been recently enriched by increasing reports of incomplete KD, mostly in children younger than 12 months, and this should be suspected in every infant showing fever for more than 5 days with documented systemic inflammation in terms of increased C-reactive protein (CRP) and white blood cell count [15–17]. The diagnosis of incomplete KD is suggested if less than 4 main clinical features are found after exclusion of many febrile illnesses. In a 2-year nationwide survey (counting more than 23.000 children with incomplete KD) 3, 2 and 1 of the main clinical features were met in 26.6, 6.1 and 0.7% of cases, respectively [18].

### Recommendation 2

Incomplete KD can be diagnosed when fever lasting more than 5 days is associated with 2 or 3 *clinical criteria*, with or without CAA.

#### Atypical KD

Atypical KD occurs in children with a classic fever pattern combined with further different non-classic signs, including aseptic meningitis, seizures, peripheral facial nerve palsy, sensorineural hearing loss, gallbladder hydrops, jaundice, acute abdomen, pancreatitis, arthritis, renal injury, orchitis, pneumonia, sterile pyuria, with or without CAA [17, 19].

### Recommendation 3

Atypical KD is diagnosed when fever lasting more than 5 days, not otherwise explained, is combined with non-classic manifestations nonresponsive to standard treatments, with or without CAA.

## The clinical course of Kawasaki disease

KD clinical course can be divided into three phases:

(a) acute (1st-2nd week), in which diagnosis should be established and treatment started,

(b) subacute (3rd-4th week), characterized by defervescence, periungual skin desquamation, thrombocytosis and eventual formation of CAA,

(c) convalescence phase (5th–8th week), in which all KD signs disappear, inflammatory markers subside to normal and nail indentations (Beau’s lines) may appear.

Child’s extreme irritability is characteristic during the acute phase of KD [20].

## Investigations in Kawasaki disease

Diagnosis of KD should be considered in all children displaying fever for 5 days (or more), as prognosis relies on a prompt treatment. Consequently, in case of suspected KD, it is important to recommend patient’s hospitalization. Unfortunately, laboratory tests are nonspecific for KD, though leukocytosis, anemia and elevation of acute-phase reactants, such as CRP, can support the diagnosis in combination with classic features [21, 22]. Hypoalbuminemia is common and associated with more severe and more prolonged acute disease. Urinalysis may show pyuria in up to 80% of children. Additional helpful findings are elevated levels of brain natriuretic peptide (BNP) and N-terminal pro-BNP. Thrombocytosis is characteristic during the subacute phase, peaking in the third week and normalizing 4-to-6 weeks after onset. Thrombocytopenia is rare, but may occur in the first 1–2 weeks of illness: it can be a sign of disseminated intravascular coagulation or macrophage activation syndrome (MAS) and is a risk factor for the development of CAA [23].

### Recommendation 4

Abnormal laboratory parameters (mostly the elevation of C-reactive protein) can *only* support the diagnosis of KD in febrile patients with evocative clinical features.

## Treatment of Kawasaki disease

### Intravenous immunoglobulin

The main goal of treatment in the acute phase of KD is suppressing systemic inflammation and minimizing the risk of developing CAA. Administration of IVIG is overall recognized as the “*first-line*” treatment for KD both in terms of efficacy and safety [24]. In particular, high-dose IVIG (2 g/kg of body weight in a single infusion) significantly reduces the incidence of CAA and overthrows both inflammation and fever, as shown by a systematic Cochrane review: IVIG is indicated in typical, incomplete and atypical KD [25]. IVIG infusion should be performed in 10–12 h if patient’s cardiac function is normal or in 16–24 h for patients displaying cardiac failure. IVIG should be administered before the 10th day of illness, best if before the 7th day since onset, as CAA might appear starting from the 8-9th day [26]. Treatment before the 5th day of fever should be reserved to exceptional cases of unequivocal diagnosis of KD [27]. IVIG should also be administered to children presenting after the 10th day of illness in case of persistent fever or ongoing systemic inflammation. Defervescence after IVIG occurs usually within 48 h in about 80–85% of cases. A reoccurring fever 36–48 h after a first administration of IVIG configures the framework of IVIG-resistant KD (this approximately occurs in 15–20% of patients) [28]. Nowadays there is no universally accepted treatment for these patients yet, and therefore the management of these cases should be established individually.

### Recommendation 5

IVIG at the dose of 2 g/kg of body weight is the treatment of choice for KD, preferably given within the 10th day, better if within the 7th day of illness, but as soon as possible after diagnosis.

### Aspirin

Treatment of KD is completed by adding aspirin (acetylsalicylic acid, ASA): medium-high dosage of ASA (from 80 mg/kg/day, mostly in the USA, to 30–50 mg/kg/day in Japan, UK and Europe, divided into 4 doses) is used for its anti-inflammatory effect during the acute phase, while 48 h after defervescence ASA can be switched to a lower dosage (3–5 mg/kg/day in a single dose) due to its anti-platelet effect. The overall duration of anti-platelet treatment is 6–8 weeks in KD patients without CAA, while it is maintained until the resolution of coronary lesions in patients with CAA [29]. Recent studies have also suggested that ASA could be started immediately at the anti-platelet dosage in the acute phase, though further randomized clinical trials are required to compare the efficacy of medium-high and low-dose aspirin [30]. Medium-high dose ASA should be replaced in case of concurrent varicella or influenza to avoid the potential development of Reye’s syndrome [31]. Conversely, low-dose ASA has not been associated with the occurrence of this syndrome: however, in case of symptoms or exposition to influenza or varicella, parents of children with KD are asked to promptly contact their physician [13]. Children may receive dipyridamole (1–5 mg/kg/day divided into 3 doses), ticlopidine (2–7 mg/kg/day divided into 2 doses) or clopidogrel (1 mg/kg/day in a single dose up to a maximum of 75 mg/day in children > 2 years or 0.2 mg/kg of body weight in children < 24 months) in replacement of ASA, even if there is less experience with the use of these drugs. Ibuprofen (like other nonsteroidal anti-inflammatory drugs involving the cyclooxygenase pathway) antagonizes the anti-platelet effect of low-dose ASA and usually should be avoided in children on anti-platelet ASA [13]. If ibuprofen or similar drugs are needed, alternative anti-platelet therapies (e.g. clopidogrel) should be considered.

### Recommendation 6

Treatment of KD is completed by ASA given at a daily dosage of 30–50 mg/kg in the acute phase of KD until 48 h after the disappearance of fever, then switched to the anti-platelet dose (3–5 mg/kg once daily).

### Recommendation 7

Low-dose ASA must be continued until 6-to-8 weeks in children without CAA and continued in children with CAA until the resolution of coronary artery lesions.

## Recurrence of Kawasaki disease

Recurrence of KD ranges from 1.4 to 3% of cases, and symptoms are often the same as for the first episode. IVIG resistance, the elevation of liver enzymes and reduced hemoglobin level are risk factors for the recurrence of KD. In addition, recurrent forms of KD can be associated with a higher incidence of CAA [32]. Recurrent KD requires the same treatment as used for the first episode. Hereditary autoinflammatory syndromes need to be considered for a comprehensive differential diagnosis of recurrent KD in childhood [33–35].

## Non-responsiveness of Kawasaki disease

Resistant KD is defined by failure to respond to IVIG therapy and consists in a recrudescent fever 36–48 h after IVIG infusion [36]. It is believed that IVIG-resistant KD reflects severity of the underlying inflammation, explaining the increased incidence of CAA in this subset of patients. Severe anemia at disease onset, early development of CAA, and signs of MAS or septic shock should be considered in predicting the risk of IVIG non-responsiveness [37]. The identification of patients with KD having a higher propensity to be IVIG-refractory is challenging: many efforts have been made to discriminate which patients might not respond to IVIG in order to undertake a more aggressive treatment combined with IVIG, but different scores useful in the Eastern Asiatic populations have been unsuccessful in the Caucasians [21, 38–40].

### Recommendation 8

Resistant KD is defined by failure in the response to IVIG and is revealed by recrudescent fever reoccurring or persisting 36–48 h after IVIG infusion.

## Treatment of resistant forms of Kawasaki disease

Several second-line treatment options are available for resistant KD, which are represented by an additional IVIG infusion, intravenous methylprednisolone pulses, infliximab and other biologic drugs. Randomized controlled trials evaluating the effectiveness of these different options apart from the second infusion of IVIG are poor [41].

### Corticosteroids

Most vasculitides are treated with corticosteroids due to their immunosuppressive and anti-inflammatory properties [42]. The use of corticosteroids as second-line treatment in KD has been hugely debated, because of differences among studies (all patients versus high-risk patients, Eastern Asiatic versus Caucasian populations). Intravenous pulses of methylprednisolone at the dose of 30 mg/kg of body weight can be provided as second-line treatment for resistant KD patients [43, 44]. Indeed, non-responder patients with KD should be managed with a second IVIG cycle and - in case of failure - with 3 pulses of methylprednisolone (30 mg/kg/day), followed by oral prednisone (2 mg/kg/day, then gradually tapered up to the resolution of symptoms and normalization of CRP). Following the observation that methylprednisolone added to the standard regimen of IVIG improved outcomes of coronary arteries in Japanese patients with severe KD [45], “high-risk” patients, i.e. children less than 12 months or those having CRP higher than 200 mg/l, severe anemia at disease onset, albumin level below 2.5 g/dl, liver disease, overt coronary artery aneurysms, macrophage activation syndrome or septic shock, may require an initial therapy with IVIG (*plus* ASA) in combination with an intravenous pulse of methylprednisolone (30 mg/kg/day). When these KD patients fail to respond to such scheme, a second IVIG cycle combined with further 3 pulses of methylprednisolone (30 mg/kg/day) *plus* ASA should be given, followed by prednisone at the initial dose of 2 mg/kg/day, then tapered up to the resolution of symptoms and normalization of CRP.

### Recommendation 9

In non-responder patients with KD treatment requires a second infusion of IVIG and - in case of failure - pulses of methylprednisolone (30 mg/kg/day) for 3 consecutive days, followed by oral prednisone (2 mg/kg/day, then gradually tapered).

### Recommendation 10

In *high-risk patients* with KD initial treatment should include:

- IVIG + single intravenous pulse of methylprednisolone (30 mg/kg/day) + low-dose aspirin (3–5 mg/kg/day).
- In case of failure treatment should be implemented with a further infusion of IVIG and three pulses of intravenous methylprednisolone (30 mg/kg/day, followed by prednisone: 2 mg/kg/day, then gradually tapered) + low-dose aspirin (3–5 mg/kg/day).

### Biological drugs

Biologics may target the presumed key-cytokines involved in KD pathogenesis, namely tumor necrosis factor (TNF)-α and interleukin (IL)-1 [46, 47]. Many considerations about their use are derived from the experience with anti-TNF or anti-IL-1 drugs on small series of KD patients. Current evidence supports the use of infliximab, a chimeric monoclonal antibody against TNF-α, as rescue therapy at a single intravenous dose of 5 mg/kg of body weight (given in 2 h) for IVIG- and corticosteroid-resistant KD patients. However, no trials have evaluated its use as adjunctive therapy in patients with early evidence of CAA [48]. There are studies confirming the effectiveness of anakinra, the recombinant IL-1 receptor antagonist blocking the natural biological activity of IL-1, in children with a refractory KD, given subcutaneously at a daily dose of 4–8 mg/kg of body weight for an overall period of 15 days or for a longer period, depending on the specific clinical scenery. Anakinra might also prevent the development of CAA [49]. The use of canakinumab, a high-affinity human monoclonal antibody targeted against IL-1β (using a single subcutaneous injection of 4 mg/kg/dose) may be also a future option for cases of IVIG-resistant and corticosteroid-resistant KD [50, 51].

### Recommendation 11

Current evidence supports the use of infliximab as rescue therapy in IVIG- and methylprednisolone-refractory patients with KD; IL-1 blockade with anakinra is highly promising in treating the most dramatically severe multi-refractory patients with KD, with potential benefits also on the cardiovascular complications.

**Fig. 1:**
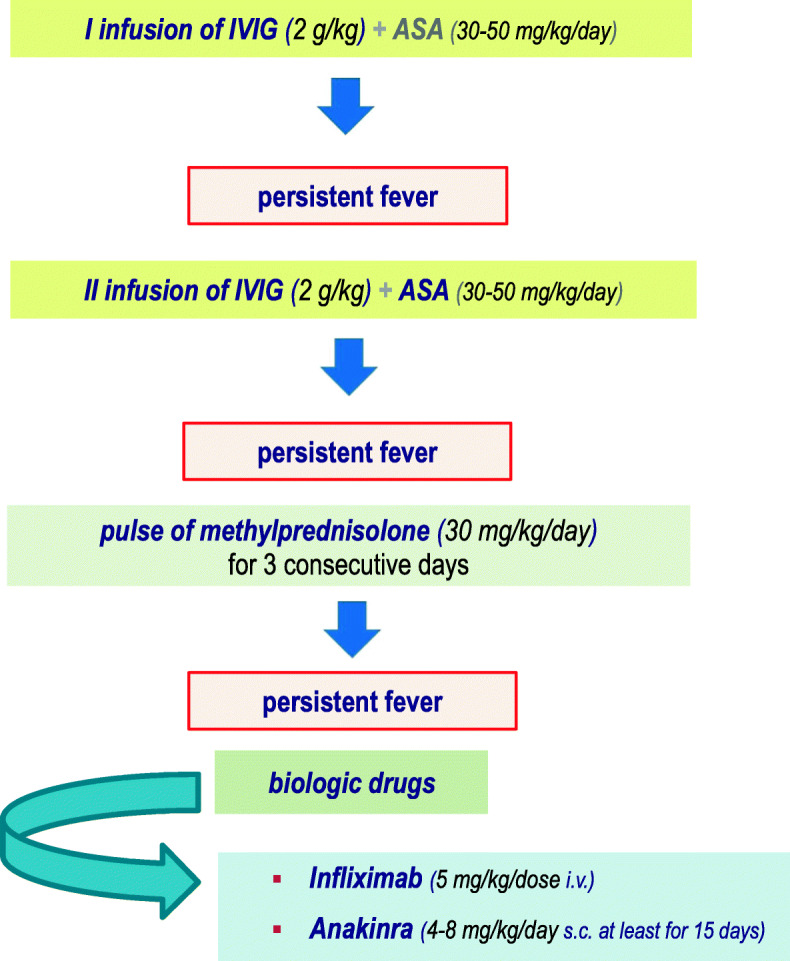
General Treatment of patients with KD

**Fig. 2:**
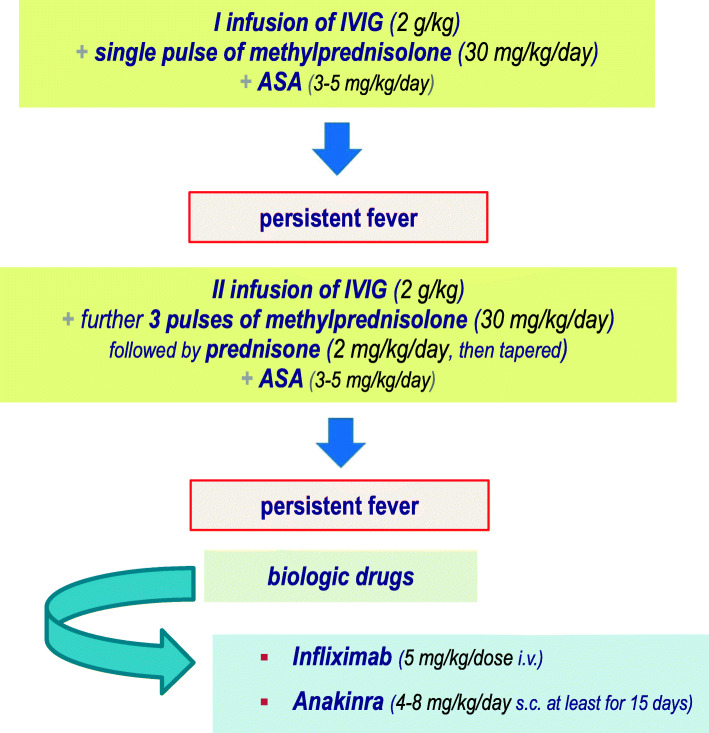
Treatment of High-Risk patients with KD. (children less than 12 months or children having C-reactive protein higher than 200 mg/l, severe anemia at disease onset, albumin level below 2.5 g/dl, liver disease, overt coronary artery aneurysms, macrophage activation syndrome or septic shock)

We have summarized the therapeutic recommendations for patients with KD (Fig. 1) and for those who should be defined as “high-risk” patients (Fig. 2).

## Complications of Kawasaki disease

The most relevant risk for children with KD is the development of vascular abnormalities, affecting small-to-mid size arteries, but characteristically the coronary arteries. A coronary artery anomaly may start as ectasia, progress to moderate dilatation (5-to-8 mm of diameter) until the formation of large aneurysms (measuring more than 8 mm). A standardized classification to measure coronary arteries and characterize CAA, comparing vessel diameters to the body surface area and measuring the standard deviation from the average in *z* units, is listed in Table 1 [52]. This classification is recommended for the right coronary, left anterior descending and left main coronary arteries, and *z*-scores with reference values for pediatric echocardiography are available at the website *www.parameterz.com*.

**Table 1:** Classification of coronary artery abnormalities in the acute phase of Kawasaki disease

No coronary artery involvement: z-score < 2	
Dilatation of the coronary artery: z-score > 2 to < 2.5 SD	
Small aneurysm of the coronary artery: z-score ≥ 2.5 to < 5 SD	
Medium aneurysm of the coronary artery: z-score ≥ 5 to < 10 SD	
Giant aneurysm of the coronary artery: z-score ≥ 10 SD	

Coronary artery involvement is virtually absent if the *z*-score is < 2 and present if the *z*-score is beyond 2*.* Patients with giant aneurysms (having *z*-scores ≥10) display the highest risk of both coronary thrombosis and stenosis [53]. Other cardiovascular complications include myocarditis, pericarditis, myopericarditis, valvular lesions, arrhythmias, heart failure in addition to coronary thrombosis or stenosis and myocardial infarction [54]. Pneumonia, encephalitis, seizures, paralytic ileus, cholecystitis, liver dysfunction, cholecystitis, anemia, hypoalbuminemia, electrolyte imbalance, diarrhea, vomiting and dehydration might occur in KD, and specific treatments may be required [19]. Other severe non-cardiovascular complications are MAS, largely known to occur in a host of disorders characterized by hypercytokinemia, heralded by non-remitting fever, impaired liver function, hyperferritinemia, hypertriglyceridemia, hypofibrinogenemia, pancytopenia and frequently hemophagocytosis [55, 56], and KD-related shock syndrome, associated with hypotension, platelet consumption and increased risk of CAA [57].

## Evaluation of the cardiovascular involvement in Kawasaki disease

KD vasculitis involves a variety of blood vessels and, in particular, small muscular arteries, being caused by diffuse neutrophil infiltration with necrotizing features, which might potentially lead to vessel dilatation mostly occurring during the subacute phase [58]. In general terms, CAA may start as ectasia which might regress within 8 weeks in most cases [59]. The regression of small or medium coronary aneurysms might occur within 1–2 years after onset, but large and giant aneurysms may persist over a long time due to local fibrosis and calcifications, with the risk of acute coronary syndrome [60]. Non-responsiveness to IVIG is a crucial sign to define KD severity, as these children have the highest risk of developing CAA [61]. Additionally, CAA are most prevalent in patients having incomplete KD [62].

### Recommendation 12

The most relevant complications in KD are cardiovascular sequelae starting as CAA, which are more frequently disclosed during the subacute phase of the disease.

### The role of echocardiography

Bidimensional and color-Doppler echocardiography must be performed when KD diagnosis is suspected, then after 2, 4 and 8 weeks in the non-complicated cases by cardiologists experienced in pediatrics [63]. However, it is important to ensure that the timing or results of the echocardiogram should not delay the initial treatment of KD during the acute phase. An initial echocardiogram in the first week of illness is typically normal and does not rule out KD diagnosis. The uncooperative irritable child may require sedation; if this is not possible, a further assessment should be repeated as soon as possible within 48 h after diagnosis. The coronary artery imaging should include the left main coronary artery, left anterior descending, left circumflex, right coronary artery (proximal, middle and distal segments), and posterior descending coronary artery. In patients with evolving CAA, follow-up assessments with echocardiography should be performed more frequently to monitor eventual increases in vessel dimensions and the potential presence of thrombosis.

### Recommendation 13

Echocardiography is pivotal for the diagnosis of coronary artery involvement in children with KD and should be periodically repeated 2, 4 and 8 weeks after onset in the uncomplicated cases.

### The role of other imaging techniques

Advanced techniques for the assessment of KD-related cardiovascular sequelae are computed tomography (CT) and magnetic resonance (MR) angiography [64]. CT scan should be used to confirm CAA, define size and morphology of CAA, identify thrombosis or occlusions, and evaluate other aneurysms, both central or peripheral, in the whole body, but its use is limited by extensive radiation exposure [65]. Cardiovascular MR angiography with gadolinium-based contrast often requires the use of general anesthesia in younger patients and can be used to confirm CAA, investigate other central or peripheral anuerysms, identify biventricular systolic function, detect areas of inducible myocardial ischemia during pharmacological stress and evaluate myocardial scars [66].

### Recommendation 14

Cardiovascular CT scan and MR angiography, where available, are important to assess persistent CAA in children with KD and monitor remodeling of either coronary or systemic arteries in the whole body.

## Treatment of the cardiovascular complications of Kawasaki disease

Long-term therapy in KD patients with CAA aims at preventing the cardiovascular acute events with reduction of both incidence and severity of acute coronary syndrome. Such therapy depends on the features and extension of CAA. In the absence of “evidence-based” studies, recommendations have been drafted by pediatric retrospective studies. Platelet activation is critical in all KD phases, therefore, in case of persistent CAA, chronic use of anti-platelet low-dose ASA (3–5 mg/kg/day) must be provided, optionally associated with other anti-platelet and/or anticoagulant and/or anti-angina drugs, according to the size of aneurysms, flow characteristics, and presence or absence of myocardial ischemic changes [67].

### Supplementary anti-platelet drugs

Persistent structural multiple or complex coronary artery aneurysms may require dual anti-platelet prophylaxis, based on low-dose ASA and clopidogrel. There is still insufficient data on the use of clopidogrel, an ADP-P_2_Y_12_ receptor inhibitor, in infants and children, for whom anti-platelet response might be different. Anti-platelet action of clopidogrel is usually achieved with lower doses (0.2 mg/kg/day) in children aged < 24 months, while older children may require higher doses (up to 1 mg/kg/day): light transmission platelet aggregometry is a common method to assess platelet function in vitro for patients using clopidogrel, though universal standards are lacking [68]. Clopidogrel may be also a valid alternative to ASA in case of ASA intolerance or resistance, varicella (either the wild type disease or vaccination against varicella) and influenza [69].

### Recommendation 15

KD patients with medium-sized coronary artery aneurysms or those with multiple and complex aneurysms require dual anti-platelet prophylaxis, based on low-dose ASA (at a single dose of 3–5 mg/kg/day) and clopidogrel (at a single dose of 0.2 mg/kg/day in children aged < 24 months and up to 1 mg/kg/day in children aged ≥ 24 months).

### Anticoagulant drugs

Anticoagulants are indicated in the case of giant aneurysms, coronary thrombosis or history of acute myocardial ischemia. Warfarin (0.05–0.12 mg/kg/day once daily, carefully adjusting the dose until reaching an international normalized ratio, INR, between 2.0 and 3.0) is the most widely used drug for long-term anticoagulation [70]. The use of low-molecular weight heparin (LMWH) can be considered in infants with KD for whom a regular assessment of INR is troublesome. In particular, enoxaparin (100 IU/kg/dose every 12 h) is a LMWH with demonstrated safety and efficacy, frequently used in pediatrics as a bridging anticoagulant regimen with low risk of thromboembolism and low risk of bleeding complications [71]. Fondaparinux, an antithrombin-dependent selective inhibitor of factor Xa, is not approved for use in children. However, in comparison to LMWH, fondaparinux has several properties which make it attractive, including a longer half-life, lack of effects on bone metabolism and reduced risk of heparin-induced thrombocytopenia. Fondaparinux (at a single daily dose of 0.1 mg/kg of body weight) has been administered to children with deep venous thrombosis without severe adverse events [72]. Novel oral anticoagulants do not require routine laboratory assessment, but there are very few data about their employment in pediatrics.

### Recommendation 16

It is reasonable to treat KD patients having complex or severe CAA with low-dose ASA associated with warfarin (keeping INR targeted at 2.0–3.0) or LMWH (if regular INR checking is difficult). Triple therapy with ASA, warfarin or LMWH and clopidogrel should be considered in KD patients with a relevant risk of thrombosis.

## Non-invasive treatment of coronary artery thrombosis

Patients with giant aneurysms of coronary arteries are at higher risk of presenting thrombosis and developing acute coronary syndrome. The goal of treatment in KD patients who show coronary thrombosis is to restore flow patency, preserve the myocardial tissue and increase patient’s survival [73]. Thrombolytic treatment with tissue-type plasminogen activator is the most commonly administered therapeutic regimen in treating fresh thrombosis within medium/large or giant coronary aneurysms. A multidisciplinary approach is needed to optimize outcome of KD patients receiving thrombolysis, that should be performed by experienced interventional radiologists or cardiologists in intensive care setting to allow rapid intervention in the case of bleeding. The ability to quickly obtain coagulation testing results for ongoing adjustments of therapy is critical for managing patients who require thrombolysis and concomitant anticoagulation. The most relevant thrombolytic drug in young patients with KD complicated by coronary artery thrombosis is the intravenous recombinant tissue plasminogen activator (rtPA), and in recent years “alteplase” has become the most widely used in children. Fresh frozen plasma (10–20 ml/kg) given before using rtPA works as a plasminogen source. Various dosing regimens have been compared, but high doses of alteplase (0.5 mg/kg, 10% infused over 1–2 min and the remainder over 60 min) are more commonly used [74]. A careful monitoring of coagulation testing every 6–12 h is mandatory to prevent bleeding (maintaining the fibrinogen level > 100 mg/dl and platelet count > 50,000/mm^3^). Low-dose thrombolytic therapy may be also associated with abciximab, a glycoprotein IIb/IIIa inhibitor, given intravenously (as a bolus of 0.25 mg/kg in 30 min, then followed by 0.125 μg/kg/minute, max: 10 μg/min, for 12 h) in the case of thrombosis with high risk of forthcoming vessel occlusion [75, 76]. Both rtPA and abciximab require to be associated with low-dose ASA and intravenous heparin (10 U/kg/hour). Coronary thrombosis should be reassessed with echocardiographic imaging during and after completion of thrombolysis.

### Recommendation 17

Recombinant tissue plasminogen activator (rtPA) is the first-choice thrombolytic drug in children with KD complicated by coronary artery thrombosis; the glycoprotein IIb/IIIa inhibitor abciximab may be used in case of thrombosis with high risk of occlusion. Both therapies require a concomitant association with low-dose ASA and intravenous heparin.

## Invasive treatment of coronary artery thrombosis

Coronary artery reperfusion by both invasive cardiac interventional procedures and cardiac surgery should be considered after unsuccessful pharmacological thrombolysis or when stenotic lesions are eventually present in selected patients. It is important to consult an adult interventional cardiologist and/or an adult cardiothoracic surgeon with experience in revascularization of patients with KD if revascularization is considered.

Cardiac procedures include intracoronary catheter-directed thrombolysis, percutaneous balloon angioplasty with or without coronary stenting and rotational atherectomy with or without coronary stenting [77]. There is no standard protocol or preferred device in pediatrics, and the use of any of these modalities often relies on physician’s experience. In order to determine the most appropriate procedure it is desirable that physicians consider patient’s body size, coronary angiography findings and intravascular ultrasound. Invasive treatment should be considered in patients with or without ischemic symptoms caused by coronary artery stenosis (≥75% of the luminal diameter), but with instrumentally demonstrated ischemia (via stress electrocardiogram or stress perfusion imaging) [78]. Coronary angioplasty procedure has a risk of vessel re-stenosis and occlusion, requiring the use of stenting or alternative procedures, such as coronary artery bypass grafting or percutaneous transluminal rotational ablation [79]. Heart transplantation can be proposed in selected cases with irreversible myocardial dysfunction [80].

### Recommendation 18

First-choice cardiologic interventional treatment in patients with KD should be chosen in the shortest time based on the specific experience of a pediatric cardiac surgery team.

## Follow-up of patients with Kawasaki disease

Patients with KD must undergo regular cardiologic evaluations with electrocardiogram and echocardiography in the long-term. For patients with large coronary aneurysms diagnosed in the first months after disease onset it is recommended to perform echocardiography weekly [81]. Cardiologic evaluations performed in the convalescence phase allow to subdivide KD patients according to their cardiovascular impairment into different risk classes and establish a personalized follow-up protocol. It is reasonable to consider KD patients worthy of close monitoring for their cardiovascular risk, evaluating and controlling blood pressure, body mass index, blood lipid profile and promoting correct lifestyles [82]. Cardiologic assessment allows to consider 5 different risk classes according to the cardiovascular sequelae and establish a personalized follow-up protocol (Table 2) [1, 2, 13].

**Table 2:** Cardiovascular risk classes in patients with Kawasaki disease

Class I	*No abnormality of coronary arteries in the various phases of the disease*
Class II	*Transient coronary artery ectasia that disappears within 8 weeks*
Class III	*Single aneurysm of small-medium caliber between + 3 and + 7 SD in one or more arteries*
Class IV	*One or more aneurysms ≥ 7 SD, including multiple and complex giant aneurysms without any obstruction*
Class V	*Coronary artery obstruction at the angiography*

### Recommendation 19

Cardiologic assessments allow to split KD patients into different risk classes according to the cardiovascular sequelae and establish a personalized follow-up protocol.

## Vaccinations in children with Kawasaki disease

Treatment of KD with IVIG does not significantly interfere with inactivated vaccines, oral attenuated live virus vaccines, live virus nasal vaccines, bacillus Calmette-Guérin vaccine or yellow fever vaccine. Subsequently, these vaccines can be safely administered at any time after IVIG administration. Conversely, it is recommended to wait 10–12 months before administration of vaccines against measles, mumps, rubella (MMR), varicella (V) and MMRV, as IVIG administration might result in reduced immunogenicity and interfere specifically with the immune response of these live attenuated vaccines. A seasonal inactivated influenza vaccine is recommended in children with KD receiving ASA [83].

### Recommendation 20

All inactivated vaccines can be safely administered at any time after IVIG in KD patients. Attenuated live virus vaccines (MMR, V and MMRV vaccines) should be administered 10–12 months after the administration of IVIG to avoid a reduced specific immune response in KD patients; influenza vaccination is recommended in KD patients receiving ASA.

## A relationship with the SARS-CoV-2 pandemic threat with Kawasaki disease?

During the 2020 pandemic caused by the novel coronavirus (SARS-CoV-2) infection a number of severely ill patients with a systemic inflammatory syndrome, which has been named “pediatric multisystem inflammatory syndrome temporally associated with coronavirus disease (COVID-19) or PIMS-TS” in the UK and “multisystem inflammatory syndrome associated with COVID-19 or MIS-C” in the USA, have been reported. The pathogenesis of this multisystem syndrome is rather unclear, though some overlapping features suggesting an immune-mediated vasculitis like KD have been described: the inflammatory response in this condition differs from KD with respect to cell subsets involved and biomarkers associated with vascular damage [84]. There has been a huge debate about the substantial risk that first-line providers encountering febrile patients might miss or delay the diagnosis of KD and proper treatment with IVIG. While there are no definite proofs of a relationship between SARS-CoV2 and KD, it is important to remind a high suspicion level for KD in all children with prolonged fever, mostly those younger than 12 months, and to perform echocardiograms to monitor eventual cardiovascular complications in these little patients even after a confirmed infection by SARS-CoV-2 [85].

## Final remarks

This list of 20 revised recommendations, drafted 3 years after the American Heart Association Statement and 2 years after the publication of the Italian Guidelines for diagnosis and treatment of KD, summarizes the most relevant clues to manage KD patients, highlighting relevant points for both clinicians and researchers. Their aim is spreading the best practice in general management of children with such a complex disorder, so that long-term morbidity and potential mortality might be prevented across the life span. We have herein refined the definition of cardiovascular and systemic complications of KD, recurrent forms of KD and treatment modalities with corticosteroids or biological agents in refractory patients. Treatment of cardiovascular complications with anti-platelet drugs and/or anticoagulants and treatment of coronary artery thrombosis with both pharmacological and non-pharmacological approaches have been also discussed. Every clinical decision-making should be specifically tailored on the individual patient with KD.

## Data Availability

No datasets were generated or analyzed during the current article.

## Abbreviations

KD: Kawasaki disease
CAA: Coronary artery abnormalities
IVIG: Intravenous immunoglobulin
ASA: Acetylsalicylic acid (aspirin)
CT: Computed tomography
MR: Magnetic resonance
CRP: C-reactive protein
TNF: Tumor necrosis factor
IL: Interleukin
INR: International normalized ratio
LMWH: low molecular weight heparin
MMR: Mumps, measles, rubella
V: Varicella
MMRV: measles, mumps, rubella, varicella
rtPA: Recombinant tissue plasminogen activator
